# Inhibition of microRNA-155 regulates gastric mucosal barrier repair and inflammation by targeting SOCS1 for the treatment of acute gastritis

**DOI:** 10.1038/s41598-025-25642-9

**Published:** 2025-11-24

**Authors:** Lei Luo, Kai Zhang, Siyu Mu, Shuangyong Liu, Wenjing Xiao, Xiaolei Liu

**Affiliations:** 1 Department of Gastrointestinal Surgery, The Affiliated Hospital of Qingdao University, Qingdao, 266075 The People’s Republic of China; 2 Department of Hepatobiliary Surgery, The Affiliated Hospital of Qingdao University, Qingdao, 266075 The People’s Republic of China; 3 Department of Thyroid Surgery, The Affiliated Hospital of Qingdao University, Qingdao, 266075 The People’s Republic of China; 4 Department of Medical Oncology, The Affiliated Hospital of Qingdao University, Qingdao, 266075 The People’s Republic of China

**Keywords:** miR-155, Inflammation, Cytokines, Acute gastritis, Ethanol-induced injury, Cytokines, Histocytochemistry

## Abstract

Acute gastritis is a common gastrointestinal disorder characterized by rapid onset of mucosal injury and infiltration of inflammatory cells, often induced by chemical irritants such as ethanol. Although inflammation is essential for mucosal defense and repair, excessive immune responses can lead to tissue damage and impaired healing. MicroRNAs (miRNAs), particularly those involved in immune regulation, have emerged as important modulators in various inflammatory conditions. However, their specific roles in the early phase of acute gastric inflammation remain unclear, and the mechanisms by which they influence mucosal damage are still not fully understood. In this study, we investigated the expression and functional significance of microRNA-155 (miR-155) in a mouse model of ethanol-induced acute gastritis. Our results showed that miR-155 expression was significantly upregulated in gastric mucosa following ethanol exposure, concomitant with elevated levels of pro-inflammatory cytokines including TNF-α and IL-6. Histological examination revealed that inhibition of miR-155 using a specific antagomir reduced inflammatory cell infiltration and attenuated mucosal injury. These findings suggest that miR-155 contributes to the exacerbation of acute gastric inflammation by promoting cytokine expression and epithelial damage. Importantly, this study highlights the role of miR-155 as an early-response regulator during acute gastric injury. Targeting miR-155 may offer a novel therapeutic strategy for the prevention or treatment of acute gastric mucosal damage. Furthermore, our findings provide a foundation for future exploration of miRNA-based interventions in inflammation-related gastrointestinal diseases.

**Supplementary Information:**

The online version contains supplementary material available at 10.1038/s41598-025-25642-9.

## Introduction

Acute gastritis is a common gastrointestinal disorder characterized by sudden onset of mucosal congestion, edema, and infiltration of inflammatory cells in the gastric lining^1^. It typically develops rapidly in response to chemical irritants such as excessive alcohol consumption, nonsteroidal anti-inflammatory drugs (NSAIDs), or bacterial toxins. These triggers disrupt the gastric epithelial barrier, leading to mucosal hemorrhage and local immune activation, accompanied by a sharp increase in pro-inflammatory cytokines, including tumor necrosis factor-α (TNF-α), interleukin-1β (IL-1β), and interleukin-6 (IL-6). While acute gastritis is often self-limiting, repeated or unresolved episodes may progress to chronic atrophic gastritis, peptic ulcers, or even gastric cancer^2,3^. Thus, understanding the early immunological events that underline mucosal inflammation is crucial for identifying novel targets for prevention and therapy.

Current therapeutic options such as proton pump inhibitors (PPIs) and H. pylori eradication primarily alleviate symptoms or target specific etiologies but do not directly modulate the immune signaling cascades involved in early mucosal inflammation. Moreover, non-infectious forms of gastritis, such as those induced by alcohol or NSAIDs, remain clinically challenging due to a lack of targeted immunoregulatory strategies. Therefore, a better understanding of the molecular events driving acute gastric inflammation is critical for the development of more precise and preventive therapies.

MicroRNAs (miRNAs) are short, non-coding RNA molecules that regulate gene expression at the post-transcriptional level, primarily through base-pairing with the 3′ untranslated regions (3′UTRs) of target mRNAs^4^. Over the past decade, their roles in shaping gastrointestinal immune responses and preserving mucosal homeostasis have gained significant research interest^5,6^. Dysregulation of miRNA expression has been implicated in several inflammatory and neoplastic diseases^7^, including inflammatory bowel disease (IBD), *Helicobacter pylori*–associated gastritis, and colorectal cancer^8,9^. These regulatory RNAs exert their effects largely through modulating classic inflammatory pathways such as NF-κB, JAK/STAT, and MAPK^10^. Among them, miR-155 has emerged as a prototypical pro-inflammatory miRNA that is rapidly induced by TLR signaling, cytokine stimulation, or microbial infection^11–13^. It promotes inflammatory responses by suppressing key negative regulators such as SOCS1^14–16^ and SHIP1^17^, thereby contributing to macrophage activation, T cell differentiation, and cytokine amplification^17–19^.

Although the role of miR-155 has been extensively studied in chronic inflammatory conditions^20,21^, its involvement in acute gastric inflammation—particularly during the early phase of chemically induced injury—remains poorly defined^22,23^. In particular, the involvement of SOCS1 as a functional target of miR-155 in acute gastric inflammation has not been well characterized. We hypothesize that miR-155 promotes acute gastric injury by downregulating SOCS1, thereby amplifying inflammatory signaling. To test this, we employed a well-established HCl/ethanol-induced mouse model of acute gastritis, alongside LPS-stimulated human gastric epithelial cells (GES-1), to investigate the expression dynamics, regulatory targets, and therapeutic potential of miR-155 inhibition. This study aims to provide mechanistic insight into the miR-155/SOCS1 axis and its potential value in modulating early mucosal.

## Material and methods

### Materials

Human gastric epithelial GES-1 cells were used as the in *vitro* model. Cells were cultured in RPMI-1640 medium supplemented with 10% fetal bovine serum and 1% penicillin–streptomycin under standard conditions. Experimental materials included miR-155 mimics and inhibitors, SOCS1 overexpression plasmids, biotinylated RNA probes, antibodies for Western blotting, ELISA kits for cytokine detection, transfection reagents, and chemical compounds used for cell stimulation or animal treatment. Detailed catalog numbers and suppliers of all reagents are listed in Supplementary Table S1.

### Cell culture

GES-1 cells were cultured in RPMI-1640 medium supplemented with 10% fetal bovine serum and 1% antibiotic solution. Cells were maintained at 37 °C in a humidified incubator containing 5% CO₂.

### RNA isolation and qRT-PCR

Total RNA was extracted from gastric tissues and GES-1 cells using a standard phenol-based method. RNA concentration and purity were assessed using a microvolume spectrophotometer. cDNA synthesis was performed with a commercial reverse transcription kit, and quantitative PCR was carried out using SYBR Green dye on a real-time PCR system. Gene expression was normalized to internal controls (U6 or GAPDH), and relative levels were calculated using the 2^ − ΔΔCt method. (All primer sequences used for qPCR are listed in Table 1.)

**Table 1:** The sequence of primer.

Primer	Sequence
MicroRNA-155 forward	5′-TTAATGCTAATCGTGATA-3′
MicroRNA-155 reverse	5′-AGTGCAGGGTCCGAGGTATT-3′
U6 forward	5′-CTCGCTTCGGCAGCACA-3′
U6 reverse	5′-AACGCTTCACGAATTTGCGT-3′
TNFα forward	5′- CCGACCACCACTACAGCAAG -3′
TNFα reverse	5′- GGGCAGGGAACCAGCATCTT -3′
IL-1β forward	5′-ATGATGGCTTATTACAGTGGCAA-3′
IL-1β reverse	5′-GTCGGAGATTCGTAGCTGGA-3′
IL-6 forward	5′-TTCGGTCCAGTTGCCTTCTC-3′
IL-6 reverse	5′-TCTTCTCCTGGGGGTACTGG-3′
IL-18 forward	5′-TCTTCATTGACCAAGGAAATCGG-3′
IL-18 reverse	5′-TCCGGGGTGCATTATCTCTAC-3′
iNOs forward	5′- CGCATGACCTTGGTGTTTGG-3′
iNOs reverse	5′- CATAGACCTTGGGCTTGCCA-3′
FOSL2 forward	5′-CAGAAATTCCGGGTAGATATGCC-3′
FOSL2 reverse	5′-GGTATGGGTTGGACATGGAGG-3′
XKR4 forward	5′-CTTGCAGCTCGGGCAAATC-3′
XKR4 reverse	5′-AGCATACTCACATCCGCATACT-3′
HBP1 forward	5′-TCATCACCATTGGAAGGAGGA-3′
HBP1 reverse	5′-TTGCACCATCCCAAATCATCA-3′
SOCS1 forward	5′-CACGCACTTCCGCACATTC-3′
SOCS1 reverse	5′-TAAGGGCGAAAAAGCAGTTCC-3′
SEMA5A forward	5′-GATCCTGCCATTTACCGAAGC-3′
SEMA5A reverse	5′-AGATGACACAAAGTTTGGCTCA-3′
CSF1R forward	5′-GGGAATCCCAGTGATAGAGCC-3′
CSF1R reverse	5′-TTGGAAGGTAGCGTTGTTGGT-3′
SGK3 forward	5′-AAAGACGAGCAGGACTAAACG-3′
SGK3 reverse	5′-GGACTGTCCATTTGAAGGAATGC-3′
S1PR1 forward	5′-AACTGACCTCGGTGGTGTTC-3′
S1PR1 reverse	5′-CTGCCAACAGGTCTGAGAGG-3′
GAPDH forward	5′-CTGGGCTACACTGAGCACC-3′
GAPDH reverse	5′-AAGTGGTCGTTGAGGGCAATG-3′

### miRNA mimics, inhibitors, and plasmid transfection

GES-1 cells were transfected with miR-155 mimic (5′-UUAAUGCUAAUCGUGAUAGGGGU-3′, 5′-ACCCCUAUCACGAUUAGCAUUA-3′), inhibitor (5′-ACCCCUAUCACGAUUAGCAUU-3′), or corresponding negative controls (5′-CAGUACUUUUUGUGUAGUACAA-3′) using a commercial lipid-based transfection reagent, according to the manufacturer’s protocol. For rescue assays, cells were co-transfected with miR-155 mimic and a SOCS1-expressing plasmid, or with an empty vector. After 48 h, cells were collected for subsequent analyses.

### Cytokine analysis (ELISA)

Culture supernatants were collected, and in cytokine levels, including TNF-α, IL-1β, IL-6, and IL-10, were quantified by enzyme-linked immunosorbent assay using commercially available kits, following standard protocols.

### Western blot analysis

GES-1 cells were lysed in RIPA buffer containing protease and phosphatase inhibitors. Protein concentrations were quantified using the BCA protein assay kit. Proteins (30 mg per lane) were separated by SDS-PAGE, transferred onto PVDF membranes, and blocked in 5% non-fat milk. Membranes were incubated overnight at 4 °C with primary antibodies, followed by incubation with HRP-conjugated secondary antibodies. Protein bands were visualized using ECL substrate. Band intensity was quantified using ImageJ software (NIH, Bethesda, MD, USA) and normalized to GAPDH expression.

### RNA pulldown assay

A biotin-labeled miR-155 probe (biotin-5′-ACCCCUAUCACGAUUAGCAUUA-3′) and a negative control probe (5′-UUCUCCGAACGUGUCACGUTT-3′). GES-1 cells were lysed in RNA binding buffer, and 1 mg of lysate was incubated with 1 μg of biotin-labeled RNA probe at 4 °C overnight. Streptavidin magnetic beads were added to pull down RNA–protein complexes. The bound fractions were analyzed by Western blot to detect SOCS1 protein enrichment.

### Animals and gastritis model

Male C57BL/6 mice (8–10 weeks old) were housed under specific pathogen-free conditions with free access to food and water. A total of 24 mice were randomly assigned to Control (saline), Model (HCl/EtOH), Ranitidine-treated, and miR-155 antagomir-treated four groups (n = 6 per group). Ranitidine was administered orally on a regular schedule at three time points: Day 0, Day 2, and Day 4, with the final dose on Day 4 given 1 h before HCl/ethanol-induced model establishment. Meanwhile, miR-155 antagomir or scrambled control antagomir was injected via the tail vein 6 h prior to model induction.

Previous reports using ethanol-induced acute gastritis models indicated that a group size of 5 animals is sufficient to detect significant differences in histological damage and cytokine expression^24,25^. To minimize potential variability associated with miR-155 modulation, the group size was increased to 6 animals. Group allocation was performed using a random number table to ensure randomization. Histological evaluation of gastric tissues (H&E staining and lesion scoring) was performed independently by pathologists from the Department of Pathology, Affiliated Hospital of Qingdao University, who were blinded to group allocation. To induce acute gastritis, mice were orally administered 0.2 mL of 150 mM HCl dissolved in 60% ethanol, while control animals received sterile saline. The study was approved by the Institutional Animal Care and Use Committee of the Affiliated Hospital of Qingdao University (Approval No. ZYFYWZLL30239). All animal experiments, including euthanasia procedures, were performed in accordance with the AVMA Guidelines for the Euthanasia of Animals (2020 Edition). For euthanasia, animals were exposed to carbon dioxide (CO₂) via a gradual displacement method in a closed chamber, with CO₂ delivered at a flow rate of 20–50% of the chamber volume per minute. This rate ensures unconsciousness occurs before CO₂ concentrations reach ~ 40% (the threshold associated with nociceptor activation), minimizing potential distress. Death was confirmed by the absence of respiratory movement, heartbeat (palpation), and pupillary reflexes; CO₂ flow was maintained for at least 1 min after respiratory arrest to ensure euthanasia completion. Subsequent tissue collection (including stomach) was performed immediately post-confirmation of death.

All methods in this study are reported in accordance with the ARRIVE guidelines^26^.

### miR-155 antagomir administration

miR-155 antagomir (5′-ACCCCUAUCACGAUUAGCAUU-3′) or scrambled control antagomir (5′-CAGUACUUUUUGUGUAGUACAA-3′) was dissolved in sterile saline and intravenously injected via tail vein at 45 mg/kg body weight (200 µL per mouse) 6 h before induce the HCl/EtOH model and sacrifice for tissue analysis.

### Histological and macroscopic evaluation

Fresh gastric tissues were dissected for histological and macroscopic evaluation. Macroscopic gastric changes were quantified using ImageJ software. The damaged mucosal area was calculated as a percentage of the total gastric mucosal area and was expressed as a percentage relative to the control group^27^.

### Statistical analysis

All data are presented as mean ± SEM from at least three independent experiments, each performed in technical triplicate. Statistical analyses were conducted using GraphPad Prism 8.0. Data normality was assessed using the Shapiro–Wilk test. For normally distributed data, unpaired t-tests or one-way ANOVA were applied; for non-normally distributed data, the Mann–Whitney U test or Kruskal–Wallis test was used. *P* < *0.05* was considered statistically significant.

## Result

### miR-155 is significantly upregulated in gastric inflammation both in vivo and in vitro

**Fig. 1:**
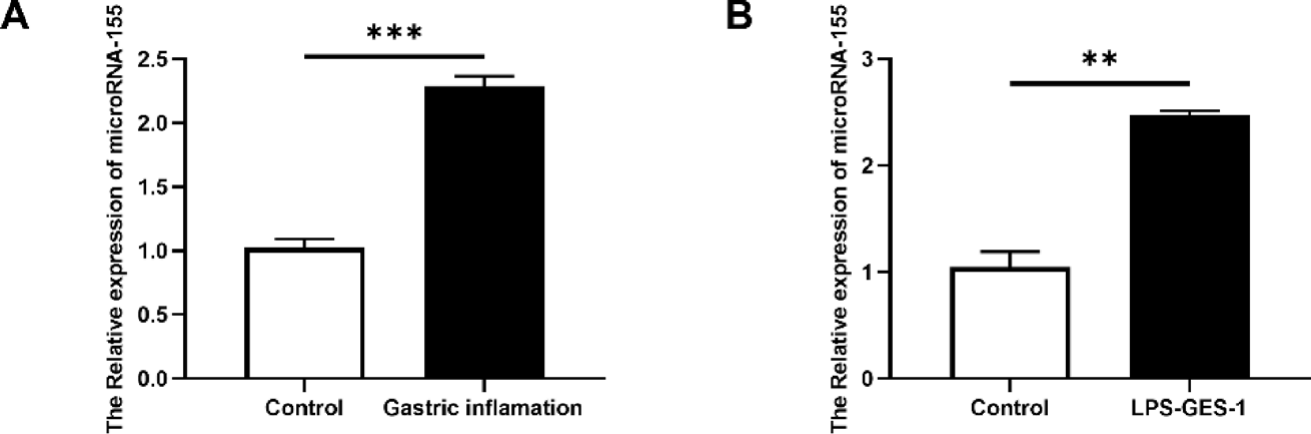
miR-155 is upregulated in acute gastric inflammation. (**A**) miR-155 expression in gastric mucosa of mice with HCl/ethanol-induced gastritis, detected by qRT-PCR. (**B**) miR-155 levels in GES-1 cells following 24 h LPS stimulation. Data are presented as mean ± SEM (n = 3); *P < 0.05, **P < 0.01 vs. control.

miR-155 is one of the most extensively studied inflammation-related microRNAs and has been shown to be upregulated in various acute and chronic inflammatory models, where it modulates immune cell activation and cytokine production. However, its expression dynamics in acute gastric inflammation—particularly during the early epithelial response—remain poorly defined. In this study, we first examined miR-155 expression in a mouse model of acute gastritis induced by HCl/ethanol. qRT-PCR analysis revealed a significant elevation of miR-155 levels in the gastric mucosa 24 h after induction compared to control mice (Fig. 1A), suggesting a potential regulatory role in the inflammatory process.

To further investigate whether similar changes occur in gastric epithelial cells, we established an in vitro model using the GES-1 cell line, a non-tumorigenic human gastric epithelial model that retains key characteristics of normal gastric mucosa and is widely used to study gastric inflammation and drug mechanisms^28,29^. These cells were treated with lipopolysaccharide (LPS, 100 ng/ml) for 24 h. Consistent with our in *vivo* findings, LPS stimulation led to a marked increase in miR-155 expression (Fig. 1B). Together, these results indicate that miR-155 is rapidly induced during the early phase of acute gastric inflammation and may play a critical role in initiating or amplifying epithelial immune responses.

### miR-155 promotes pro-inflammatory cytokine expression in gastric epithelial cells

**Fig. 2:**
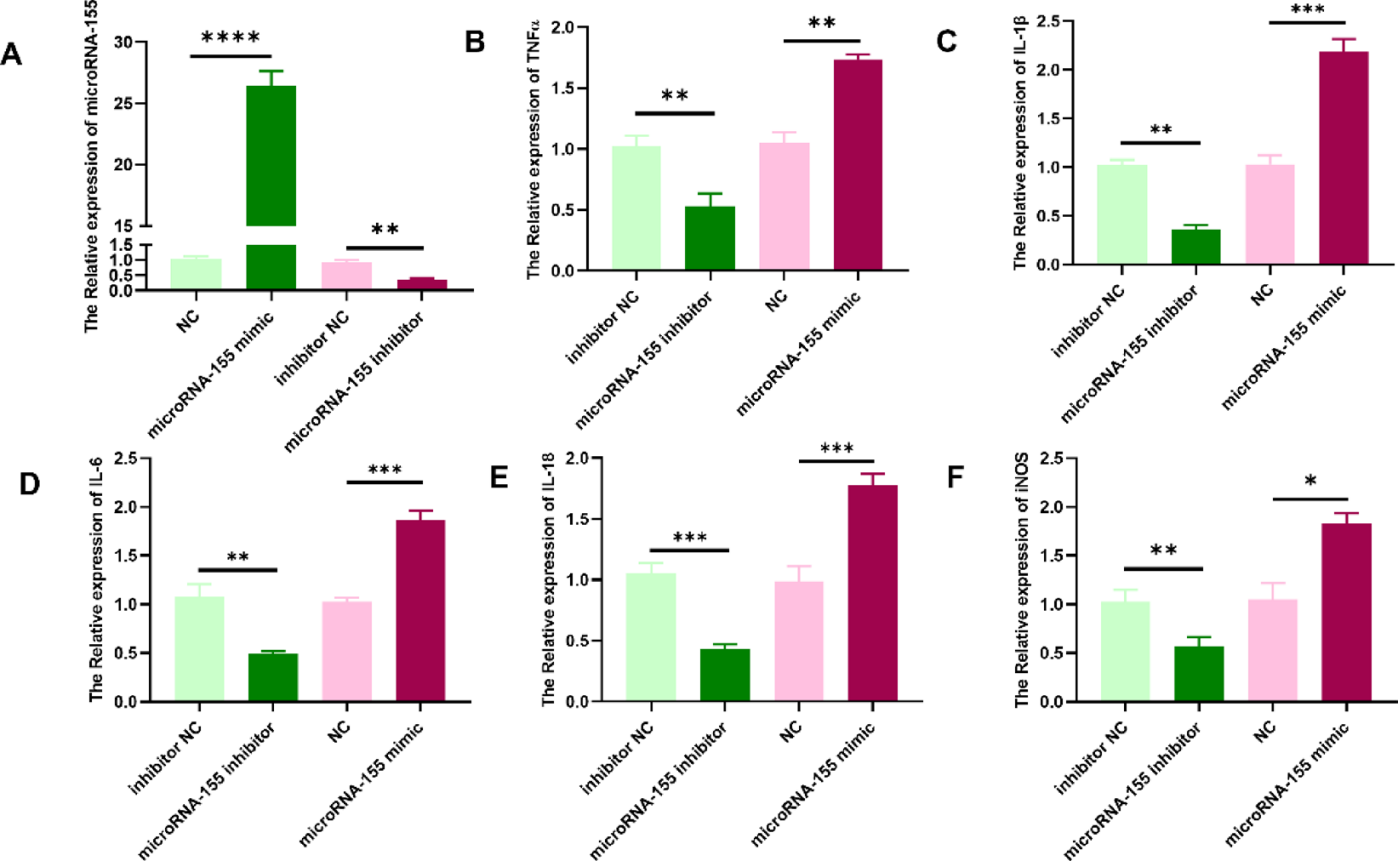
miR-155 modulates pro-inflammatory gene expression in GES-1 cells. (**A**) miR-155 levels following mimic or inhibitor transfection. (**B**–**F**) Relative mRNA expression of TNF-α (**B**), IL-1β (**C**), IL-6 (**D**), IL-18 (**E**), and iNOS (**F**). Data are presented as mean ± SEM (n = 3). *P < 0.05, **P < 0.01, ***P < 0.001, ****P < 0.0001 compared with negative control (NC) groups.

The functional role of miR-155 in gastric epithelial inflammation was further explored by transfecting GES-1 cells with a miR-155 mimic or inhibitor. qRT-PCR analysis confirmed successful transfection, with miR-155 expression markedly increased in the mimic group and significantly reduced in the inhibitor group (Fig. 2A). Functionally, overexpression of miR-155 significantly elevated the mRNA levels of several key pro-inflammatory cytokines and mediators, including tumor necrosis factor-alpha (TNF-α; Fig. 2B), interleukin-1 beta (IL-1β; Fig. 2C), interleukin-6 (IL-6; Fig. 2D), interleukin-18 (IL-18; Fig. 2E), and inducible nitric oxide synthase (iNOS; Fig. 2F). Conversely, miR-155 inhibition led to a marked reduction in the expression of these genes compared with the respective negative control groups.

These findings indicate that miR-155 functions as a positive regulator of inflammatory gene expression in gastric epithelial cells, potentially contributing to the amplification of inflammatory responses during acute gastritis.

### miR-155 enhances LPS-induced cytokine production and activates NF-κB signaling in gastric epithelial cells

**Fig. 3:**
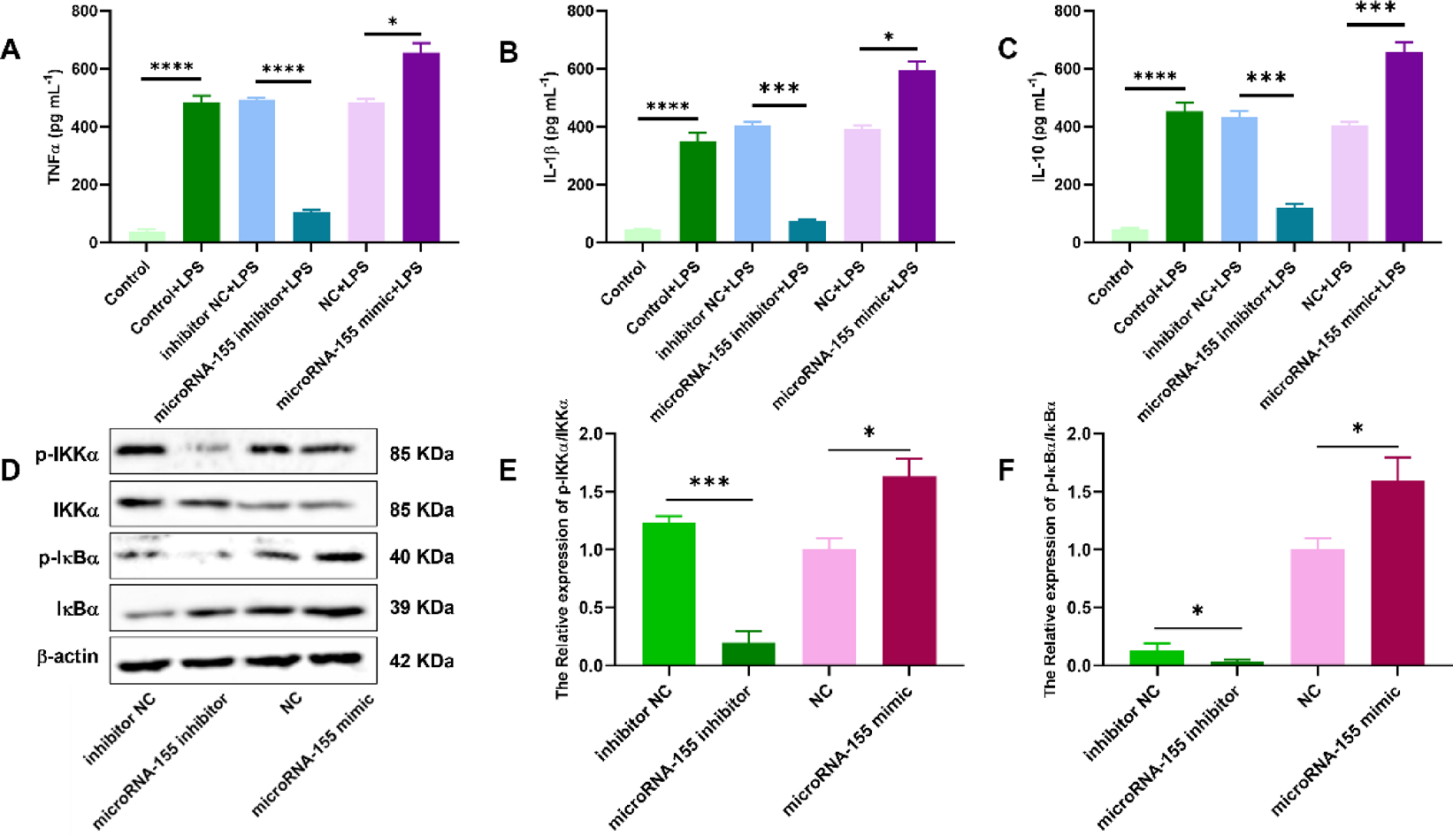
miR-155 enhances LPS-induced cytokine secretion and NF-κB signaling. (**A**–**C**) ELISA analysis of TNF-α, IL-1β, and IL-10 levels in GES-1 cells. (**D**) Western blot showing phosphorylation of IKKα and IκBα. (**E**–**F**) Quantitative analysis of p-IKKα/IKKα and p-IκBα/IκBα. Data are presented as mean ± SEM (n = 3), *P < 0.05, **P < 0.01 vs. control.

To further investigate the mechanism by which miR-155 promotes inflammation in gastric epithelial cells, we assessed its effect on cytokine secretion and NF-κB signaling in LPS-stimulated GES-1 cells. ELISA analysis revealed that transfection with miR-155 mimic significantly enhanced the secretion of TNF-α, IL-1β, and IL-10 in response to LPS stimulation compared to the negative control group (Fig. 3A–C). In contrast, the inhibition of miR-155 markedly reduced the levels of these cytokines following LPS exposure. We also examined the phosphorylation status of key components in the canonical NF-κB pathway. Western blot analysis showed that miR-155 overexpression increased the phosphorylation of IKKα and IκBα, whereas miR-155 inhibition suppressed these phosphorylation events (Fig. 3D–F; original data available in Supplementary Fig. S1). Together, these results suggest that miR-155 enhances LPS-induced inflammatory responses in gastric epithelial cells, at least in part by activating the NF-κB signaling pathway.

### SOCS1 is a direct functional target of miR-155

**Fig. 4:**
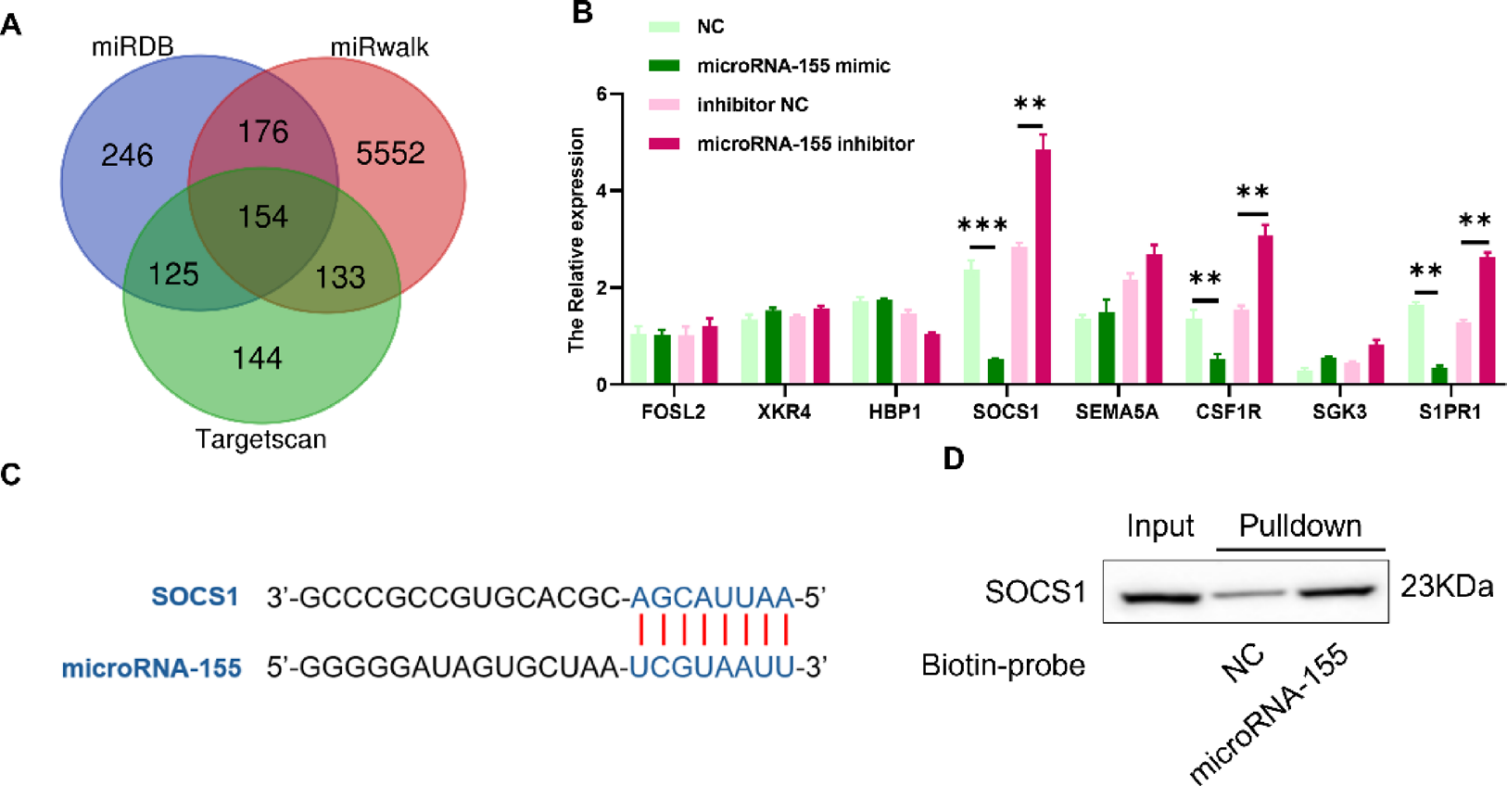
SOCS1 is a direct target of miR-155. (**A**) Venn diagram of predicted targets from three databases. (**B**) qRT-PCR of SOCS1 mRNA after miR-155 modulation. (**C**) Predicted binding site between miR-155 and SOCS1 3′UTR. (**D**) RNA pulldown assay showing SOCS1 enrichment with wild type but not mutant 3′UTR. Data are presented as mean ± SEM (n = 3). **P < 0.01, ***P < 0.001.

We next performed integrative bioinformatic analysis using the miRDB, TargetScan, and miRWalk databases to identify potential downstream targets of miR-155. A total of 154 overlapping candidate genes were identified across all three platforms (Fig. 4A). Based on their known involvement in negative regulation of NF-κB signaling, SOCS1^30^, FOSL2^31^, XKR4, and HBP1 were selected for further validation^32^.

qRT-PCR analysis revealed that miR-155 mimic transfection significantly reduced SOCS1 mRNA expression in GES-1 cells, while miR-155 inhibition restored SOCS1 levels (Fig. 4B). In contrast, the expression of the other predicted targets showed no significant changes, supporting the specificity of the miR-155–SOCS1 interaction. Sequence alignment analysis further predicted a conserved binding site for miR-155 within the 3′ untranslated region (3′UTR) of SOCS1 (Fig. 4C). To confirm the direct interaction between miR-155 and SOCS1 mRNA, an RNA pulldown assay was performed using a biotin-labeled miR-155 probe. GES-1 cells were lysed after co-transfection with either wild-type or mutant SOCS1 3′UTR constructs, and lysates were incubated with streptavidin-conjugated magnetic beads to capture the probe-associated complexes. SOCS1 protein was markedly enriched in the pulldown complex when co-transfected with wild-type 3′UTR, but not with the mutant lacking the predicted binding site (Fig. 4D). These results provide strong evidence that SOCS1 is a direct and functional target of miR-155 in gastric epithelial cells.

### miR-155 promotes inflammatory cytokine expression through SOCS1 suppression in GES-1 cells

**Fig. 5:**
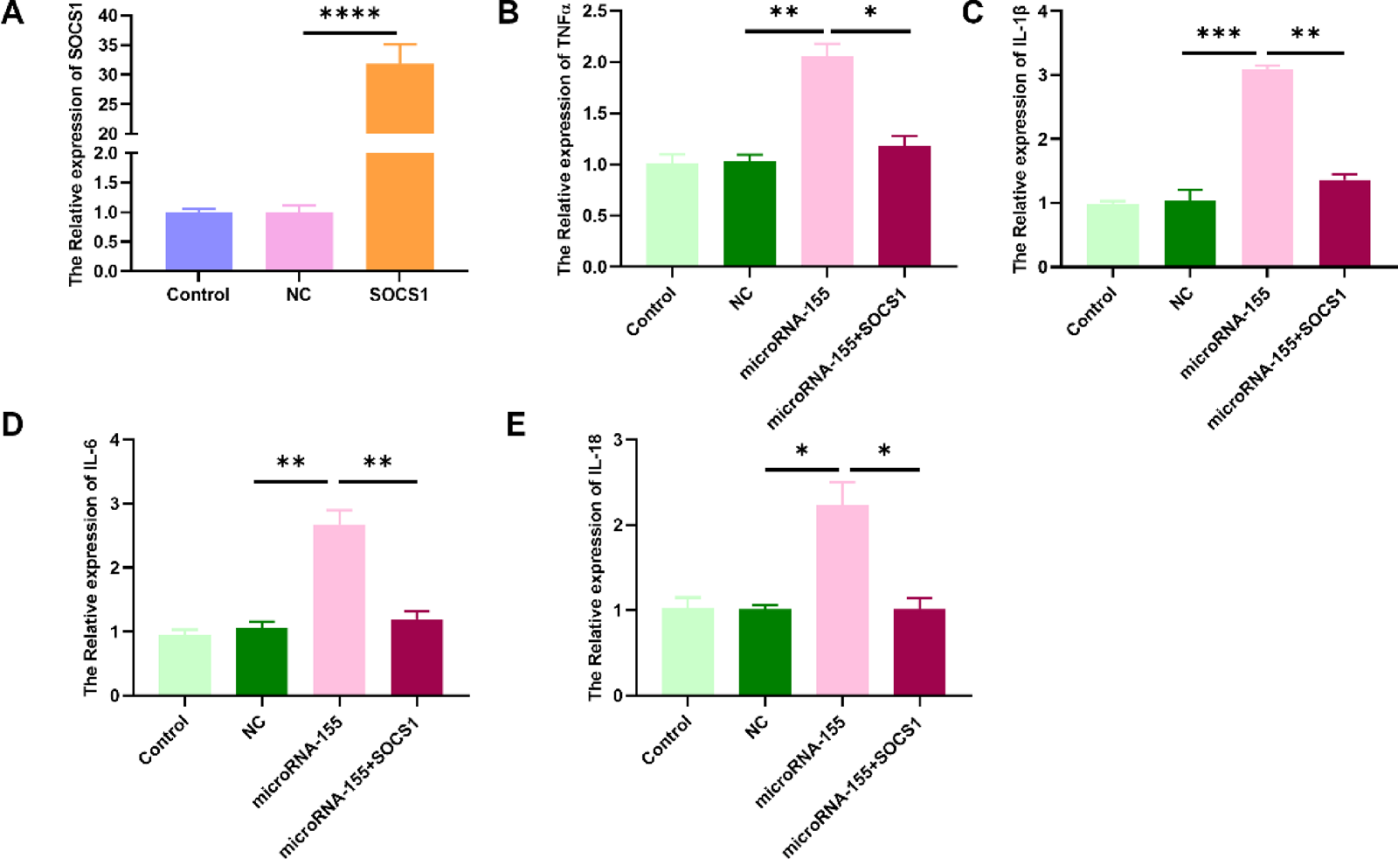
SOCS1 overexpression reverses miR-155-induced cytokine expression. (**A**) qRT-PCR validating SOCS1 plasmid overexpression. (**B**–**E**) Rescue of TNF-α, IL-1β, IL-6, and IL-18 expression by SOCS1 in GES-1 cells co-transfected with miR-155 mimic. Data are presented as mean ± SEM (n = 3), *P < 0.05, **P < 0.01.

To evaluate whether the pro-inflammatory effects of miR-155 are mediated through suppression of SOCS1, we conducted a rescue experiment in GES-1 cells. Overexpression of miR-155 markedly increased mRNA levels of TNF-α, IL-1β, IL-6, and IL-18 (Fig. 5B–E). However, these effects were substantially reversed by co-transfection with a SOCS1-expressing plasmid, which restored cytokine expression to near basal levels. qRT-PCR confirmed successful SOCS1 overexpression (Fig. 5A). These findings indicate that miR-155 enhances inflammatory cytokine expression in gastric epithelial cells, at least in part, by downregulating SOCS1. The reversal of miR-155-mediated effects by SOCS1 restoration further supports the functional relevance of the miR-155/SOCS1 regulatory axis in epithelial inflammation.

### Inhibition of miR-155 alleviates gastric inflammation in *vivo*

**Fig. 6:**
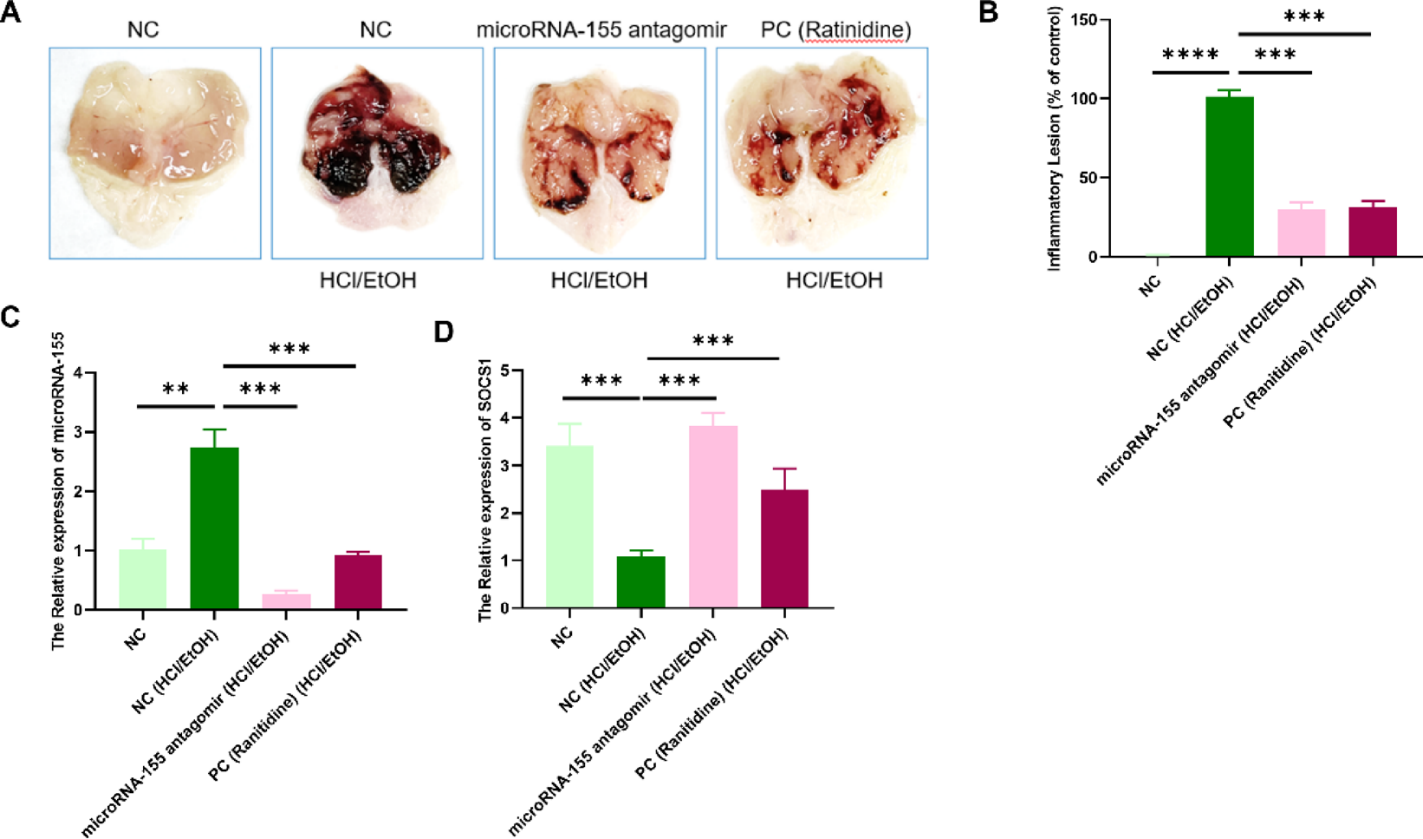
miR-155 antagomir alleviates gastric injury in *vivo*. (**A**) Gross gastric morphology in mice treated with miR-155 antagomir. (**B**) Quantification of gastric lesion scores. (**C**, **D**) Relative expression of miR-155 and SOCS1 in gastric tissues as measured by qRT-PCR. Data are presented as mean ± SEM (n = 6), *P < 0.05, **P < 0.01 vs. NC.

In order to further evaluate the therapeutic potential of targeting miR-155 in *vivo*, we employed a well-established ethanol/HCl-induced acute gastritis mouse model. Mice administered a miR-155 antagomir via tail vein injection. Remarkably, systemic delivery of the antagomir significantly alleviated gastric mucosal hemorrhage and tissue injury, producing therapeutic effects comparable to those of ranitidine (Fig. 6A). Gastric lesion scores were also markedly improved following treatment (Fig. 6B). At the molecular level, gastric tissues from antagomir-treated mice exhibited a notable reduction in miR-155 expression along with restored SOCS1 levels (Fig. 6C, D), confirming the protective effect of systemic miR-155 inhibition against acute gastric injury.

## Conclusion

In this study, we demonstrated that miR-155 expression was markedly elevated in ethanol-induced acute gastritis mice and LPS-stimulated gastric epithelial cells. Functional assays confirmed that miR-155 promotes inflammatory cytokine production, NF-κB pathway activation, and gastric mucosal injury, suggesting a critical pro-inflammatory role during acute gastric inflammation.

Previous reports established miR-155 as a potent inflammatory mediator in chronic colitis and H. pylori–associated gastritis through targeting negative regulators such as SOCS1^12^. Consistent with these findings, we identified SOCS1 as a direct target of miR-155 in gastric epithelial cells. Suppression of SOCS1 by miR-155 likely amplifies NF-κB signaling, thus exacerbating the inflammatory response. Notably, while miR-155 has traditionally been studied in immune cells, our data highlights its functional importance in epithelial cells, indicating a broader role in gastric mucosal inflammation. In *vivo*, intravenous administration of miR-155 antagomir significantly alleviated gastric injury, underscoring its therapeutic potential. While intravenous delivery ensures antagomir stability, it might limit direct mucosal targeting, suggesting future studies could explore protective oral delivery systems.

Our study has limitations, including potential undiscovered miR-155 targets, reliance on a single inflammatory stimulus, and absence of temporal profiling. Future research should address these aspects to clarify the comprehensive role of miR-155 in gastric inflammation. Collectively, these findings establish miR-155 as a key mediator in acute gastric inflammation and support miR-155 inhibition as a promising therapeutic strategy.

## Discussion

In summary, our study demonstrates that miR-155 is significantly upregulated in both in *vivo* and in *vitro* models of acute gastric inflammation, functioning as a key pro-inflammatory regulator. By promoting the expression of cytokines such as TNF-α and IL-6 and enhancing NF-κB pathway activation, miR-155 exacerbates gastric mucosal damage and inflammatory cell infiltration. Mechanistic investigations confirmed SOCS1 as a direct functional target of miR-155, whose suppression attenuates the negative regulation of inflammatory signaling, thus amplifying the inflammatory response.

Our findings align with previous studies that have described miR-155 as a critical modulator of immune responses in chronic inflammatory diseases, including chronic gastritis and inflammatory bowel disease^20,21^. However, its role in acute gastric injury—particularly during the early immunological phase—has remained poorly defined. Here, we extend the understanding of miR-155 to acute inflammation by providing both in *vitro* and in *vivo* evidence that its upregulation contributes to early mucosal injury. Notably, we identify SOCS1 as a key intermediary in this process, adding to current knowledge on miR-155–SOCS1 interactions in gastric epithelial cells and expanding the mechanistic insight into how miR-155 modulates NF-κB signaling.

The integration of animal and cell-based experiments in this study provides strong mechanistic support. miR-155 expression was consistently elevated upon inflammatory stimulation in GES-1 cells and mouse gastric tissue, accompanied by downregulation of SOCS1 and upregulation of pro-inflammatory cytokines. Inhibition of miR-155 in both models restored SOCS1 expression and attenuated inflammatory responses, indicating that the regulatory axis is conserved across systems and biologically relevant.

Importantly, intravenous administration of miR-155 antagomir effectively alleviated gastric mucosal injury in a mouse model of acute gastritis, supporting the translational potential of miR-155 inhibition as a therapeutic strategy^23,33^ and underscore the importance of early intervention in chemical-induced mucosal injury. Compared with conventional clinical strategies such as proton pump inhibitors (PPIs) or Helicobacter pylori eradication—which primarily aim to neutralize acid or eliminate infection—miR-155 inhibition represents a novel immunomodulatory approach that directly targets the molecular pathways underlying mucosal inflammation. Such nucleic acid-based therapies may complement existing treatments, especially in non-infectious or treatment-refractory cases. Nevertheless, several limitations should be acknowledged. This study was primarily preclinical and did not include patient-derived data or clinical tissue validation. The absence of human correlative datasets (e.g., miR-155/SOCS1 expression profiles in acute gastritis patients) limits direct extrapolation of our findings to clinical settings. Additionally, although our experiments demonstrate a functional relationship between miR-155 and SOCS1, other potential targets of miR-155 in the gastric epithelium remain to be explored. Future work involving patient samples, large-scale transcriptomic analysis, and extended therapeutic evaluation in chronic models will be essential to confirm the clinical relevance of our findings. In particular, we aim to collect gastric biopsy specimens from acute gastritis patients and assess miR-155/SOCS1 expression patterns, as well as explore relevant public datasets to evaluate clinical correlations.

Together, this study expands our understanding of molecular events regulating acute gastric inflammation and highlights miR-155 as a promising candidate for therapeutic intervention. Further exploration of its temporal dynamics and regulatory network may provide deeper insights into gastrointestinal immunity and epithelial repair mechanisms.

## Supplementary Information

Below is the link to the electronic supplementary material.

Supplementary Material 1

## Data Availability

The data used and/or analyzed during the current study are available from the corresponding authors upon reasonable request.
