# Supplementary material for: Inhibition of microRNA-155 regulates gastric mucosal barrier repair and inflammation by targeting SOCS1 for the treatment of acute gastritis
Source: Sci Rep. 2025 Nov 24;15:41631. doi: 10.1038/s41598-025-25642-9 (PMC12644493; doi:10.1038/s41598-025-25642-9)
Supplement: Supplementary file 1 — Supplementary Material 1 [file 41598_2025_25642_MOESM1_ESM.pdf]

Supplementary Table S1. Catalog Numbers and Suppliers of Reagents

Reagent/Item	Supplier	Catalog Number
RPMI-1640 medium	Gibco	C11875500BT
FBS (Fetal Bovine Serum)	HyClone (Cytiva)	SH30071.03
Trypsin-EDTA	Gibco	25200056
Penicillin-Streptomycin	Gibco	15140122
PBS	Sigma-Aldrich	P3813
Lipopolysaccharide (LPS)	Sigma-Aldrich	L2880
GES-1 cells	ATCC	AC-2546H
miR-155 mimic/inhibitor/controls	RiboBio	Custom synthesized
miR-155 antagomir	RiboBio	Custom synthesized
SOCS1 overexpression plasmid	YouBio	Custom synthesized
TRIzol reagent	Invitrogen	15596026
Lipofectamine 3000	Invitrogen	L3000008
SYBR Green PCR Master Mix	Acmec	AC17096
qPCR primers	Sangon Biotech	Custom Oligos
ELISA kit: TNF-α	MultiSciences	EK182HS
ELISA kit: IL-1β	MultiSciences	EK101B
ELISA kit: IL-6	MultiSciences	EK106
ELISA kit: IL-10	MultiSciences	EK110
p-IKKα antibody	Cell Signaling Technology	2697
IKKα antibody	Cell Signaling Technology	2682
p-IκBα antibody	Cell Signaling Technology	2859
IκBα antibody	Cell Signaling Technology	92242
GAPDH antibody	Proteintech	60004-1-Ig
HRP-conjugated secondary antibody	Cell Signaling Technology	7074 or 7076
Biotin-labeled miR-155 probe	Sangon Biotech	Custom synthesized
Streptavidin magnetic beads	Thermo Fisher Scientific	88817
RIPA buffer	Thermo Scientific	89900

Protease/phosphatase inhibitors	Thermo Scientific	78440
BCA Protein Assay Kit	Beyotime	P0010
PVDF membrane	Millipore	IPVH00010
ECL substrate	Millipore	WBKLS0500
Ranitidine	Solarbio	R8150

Supplementary Figure S1. WB Original Blots

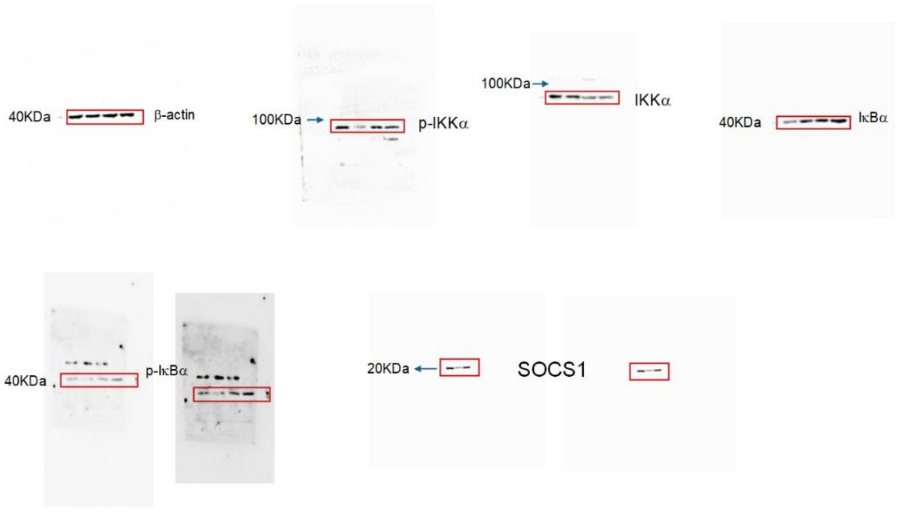

Fig S1 WB protein band
